# Health: redefined

**DOI:** 10.11604/pamj.2018.30.292.15436

**Published:** 2018-08-24

**Authors:** Obinna Ositadimma Oleribe, Omole Ukwedeh, Nicholas Jonathan Burstow, Asmaa Ibrahim Gomaa, Mark Wayne Sonderup, Nicola Cook, Imam Waked, Wendy Spearman, Simon David Taylor-Robinson

**Affiliations:** 1 Excellence and Friends Management Care Centre (EFMC), Dutse Abuja FCT, Nigeria; 2 Liver Unit, Department of Surgery and Cancer, St. Mary’s Hospital Campus, Imperial College London, Praed Street, London, W2 1NY, United Kingdom; 3 National Liver Institute, Menoufiya University, Shbeen El Kom, Egypt; 4 Division of Hepatology, Department of Medicine, Faculty of Health Sciences, University of Cape Town and Groote Schuur Hospital, Cape Town, South Africa

**Keywords:** Health promotion, health economics, social well-being

## Abstract

For many years the definition of 'health' has remained unchanged as a narrow concept, encompassing physical wellbeing from a medical context. This somewhat focused definition has attracted criticism from individuals and professional bodies alike. Recent attempts have been made to redefine health, each offering an alternative viewpoint from sociological, environmental, societal and economic standpoints. We summarize and contextualize these definitions and provide an alternative, new, all-encompassing definition of health.

## Essay

Over the past years, different healthcare professionals, whether groups or individuals, have developed and defended various definitions of what a true state of “health” actually means. Despite several different definitions, there are still debates and disagreements on the precise meaning of health. In this review, the notion of “health” is discussed, reviewed and redefined.

**What is “health”?**: Health is an amorphous word that lacks a single definition. To some, “health is wealth” -given that in the absence of good health, an individual or society cannot attain its full potential. Mahatma Gandhi validated this in 1948 when he said: “It is health that is real wealth and not pieces of gold and silver [1].” He was alluding to the idea that health is more important than monetary wealth, and that a society cannot prosper unless its people are healthy. Health is necessary for productivity and to fully enjoy life. Health is relative and has situational, professional and even societal definitions. For example, to an athlete, health may mean being physically fit so as to complete a 5000m race. For an employee forced to miss work due to a bout of influenza, health may mean being able to return to work. In contrast to physical health, a person in an unhappy relationship may be concerned about their mental health. Finally, references to “health” are used in a non-medical context. For example: “efforts to build a healthy economy” or a sports team having a “healthy starting line-up.” With so many possible applications of the word, the question arises as to what it actually means to be healthy. The word “health” is derived from an old English word, “hale”, which means “wholeness, being whole or sound.” Despite its origins, there are several etymological meanings and these definitions have evolved over time. Early definitions focused on biomedical aspects: health was seen as the ability of the body to carry out its biological functions and any disruption in these functions was viewed as a disease. For instance, the Oxford online dictionary (2016), defines health as: “a state of being, free from illness or injury [2].” Similarly, Merriam-Webster online dictionary (2016) defines health simply as: “the condition of being well or free from disease [3].” That is, the condition of being sound in body, mind, or spirit; especially freedom from physical disease or pain [3]. While these definitions have merit, their scope is limited. Perhaps the most established modern day definition of health was termed by the World Health Organization (WHO) in 1948, when it stated: “Health is a state of complete physical, mental and social well-being and not merely the absence of disease or infirmity [4].” For the first time, domains of health beyond merely biomedical aspects were considered, emphasizing the importance of mental and social wellbeing.

**Criticisms of the current definition, and existing alternatives**: However, although comprehensive, the WHO definition of health has received criticism [5,6]. Most criticisms center on the word “complete”, which many believe to be absolute, and difficult to measure. Furthermore, questions arise over whether it is even possible for a person to be without any physical, mental or social challenges. Smith argues that this prerequisite for completeness would mean many would be unhealthy most of the time [7]. In addition, the increase in the prevalence of chronic disease would mean that many with even minor long-term ailments would be persistently classified as being ill [8]. Indeed, this need for “complete” wellbeing brings the risk of over-medicalization; redefining and treating conditions not previously identified as health problems, leading to individuals receiving unnecessary interventions [8]. In 1982, Stokes, Noren and Shindell took the concept further by defining health as: “a state characterized by anatomic, physiologic, and psychological integrity; an ability to perform personally valued family, work, and community roles; an ability to deal with physical, biologic, psychological, and social stress [9].” Thus, for one to be healthy, one needs to be in perfect physical, psychological and social state. Interestingly, this definition introduces the concept of resilience, whereby health is not an absolute state of well-being as previously described [4], but also a means of coping with stressors experienced by an individual [9]. It therefore follows, that health is determined by a person's physical, psychological, social, religious and economic environment. For instance, an individual living in a comfortable and safe environment, with clean running water and healthy food, is more likely to experience good health than someone who does not have access to such amenities. A child raised in a hostile environment is more likely to develop emotional problems later in life [10]. A soldier returning from conflict may carry psychological trauma in the form of post-traumatic stress disorder.

Studies have also shown that people with low socio-economic status have increased mortality, because they are more likely to adopt detrimental lifestyles, such as smoking, alcohol and poor dietary habits [11]. The presence of family and friends may also influence health, aiding or even hindering recovery from illness. With increasing globalization, family members may find themselves living countries apart, thus unable to help one another in times of need. Furthermore, vast socioeconomic disparities both inter-country and inter-community, discrepancies between private and public healthcare services, limited healthcare work forces and geopolitical strife further contribute to disparities in individual health. On account of the limitations in the WHO and various other definitions of health, several groups have called for the review of the WHO definition of health [8]. At a Health Council of the Netherlands conference in 2009, experts argued that health was not static, but dynamic. In the conference, their preferred notion of health was: “the ability to adapt and to self-manage [12].” This concept viewed health as a dynamic process and a resource for everyday life, not merely an object to be obtained for the sake of itself, in keeping with the definition provided by Stokes, Noren and Shindell [9]. Thus to be healthy, one requires the capacity to maintain homeostasis and recover from insults [13]. This definition encompasses the ability to handle stress, to acquire skills and to maintain relationships. This ability is known as resilience, without which it would be difficult to remain healthy. Clearly, health is a complex and multifaceted term, and further attempts have been made to define it by individuals and professions alike. For instance, Suresh Vatsyayann, in 2013, saw health as: “an ever-evolving state of mind, body, and relationships perceived by an individual, a family, a group or a community for self in a particular time, space and context [14].” It can, therefore, be seen as one's ability to live his dream [15] or a person's mental or physical condition [2]. We agree with Kaila that true health is the intersection of one's physical, mental, emotional, social, economic, and spiritual state of being at any one time [16]. The aforementioned shortcomings of the WHO's definition of health, coupled with ever-more encompassing and complex descriptions of health, necessitate the need for a new, single, universal definition of health to replace the outdated one created over 60 years ago.

**Redefining health**: To develop this comprehensive acceptable definition of health, the following questions must be considered: Is health a state of complete well-being? Is a healthy person someone whose body is free from disease and able to carry on normal activities without fatigue [4]? Or is health a dynamic condition, encompassing resilience to stressors and recovery from insults in order to maintain an inner equilibrium or homeostasis [9,12,13]? Can the person with severe rheumatoid arthritis, who, with the help of therapy is able to carry out their daily routine, be said to be healthy? Or the university student, recently diagnosed with mental illness, who, after therapy, is able to return to their studies? Finally, what about the teenager diagnosed with Huntington's disease, who is currently symptom-free and able to function normally, but will not remain so forever? Can they be said to be healthy? Whatever definition is developed or adapted, to attain health, people have to draw from the resources available in the community. In other words, one cannot be healthy if their society is unhealthy. That is why, according to Public Health Agency of Canada, health is: “a positive concept that emphasizes social and personal resources, as well as physical capacities [17].” Sadly, today, the world is unhealthy. People's attitudes and habits, combined with an ever-ageing population that may not follow a healthy lifestyle, are making it more unhealthy, and events in the news can be personally disturbing. People involved in making the world an unhealthy place through acts of omission or commission like terrorism, manslaughter, kidnapping and other social vices cannot be said to be healthy, irrespective of their physical, mental, economic, emotional, or even spiritual condition. This is why we suggest redefining health as: “a satisfactory and acceptable state of physical (biological), mental (intellectual), emotional (psychological), economic (financial), and social (societal) wellbeing.” This state would result in maximum productivity, positive contributions and relevant existence in a degenerating and decaying world. It is the state of having the overall physical, mental, emotional, and social abilities to add values not just to one's self, but to society, resulting in the development of a better and sustainable world where things work, people live in harmony and community existence is enhanced.

## Conclusion

Our definition is all encompassing. We believe that this definition will help answer some of the common questions raised on health, resolve most of the current debates on the meaning of health, and help expand the meaning of health and the functions of healthcare workers to include services outside the current health ecosystem.

## Competing interests

The authors declare no competing interests.
